# Catastrophic NAD^+^ Depletion in Activated T Lymphocytes through Nampt Inhibition Reduces Demyelination and Disability in EAE

**DOI:** 10.1371/journal.pone.0007897

**Published:** 2009-11-19

**Authors:** Santina Bruzzone, Floriana Fruscione, Sara Morando, Tiziana Ferrando, Alessandro Poggi, Anna Garuti, Agustina D'Urso, Martina Selmo, Federica Benvenuto, Michele Cea, Gabriele Zoppoli, Eva Moran, Debora Soncini, Alberto Ballestrero, Bernard Sordat, Franco Patrone, Raul Mostoslavsky, Antonio Uccelli, Alessio Nencioni

**Affiliations:** 1 Department of Experimental Medicine, Section of Biochemistry, University of Genoa, Genoa, Italy; 2 Advanced Biotechnology Center, Genoa, Italy; 3 Department of Neurosciences, Ophthalmology and Genetics, University of Genoa, Genoa, Italy; 4 Laboratory of Immunology, Department of Translational Oncology, National Institute for Cancer Research, Genoa, Italy; 5 Department of Internal Medicine, University of Genoa, Genoa, Italy; 6 Department of Medicine, Massachusetts General Hospital Cancer Center, Harvard Medical School, Boston, Massachusetts, United States of America; 7 Laboratory of Glycochemistry and Asymmetric Synthesis, Swiss Federal Institute of Technology (EPFL), Batochime, Lausanne, Switzerland; University of North Dakota, United States of America

## Abstract

Nicotinamide phosphoribosyltransferase (Nampt) inhibitors such as FK866 are potent inhibitors of NAD^+^ synthesis that show promise for the treatment of different forms of cancer. Based on Nampt upregulation in activated T lymphocytes and on preliminary reports of lymphopenia in FK866 treated patients, we have investigated FK866 for its capacity to interfere with T lymphocyte function and survival. Intracellular pyridine nucleotides, ATP, mitochondrial function, viability, proliferation, activation markers and cytokine secretion were assessed in resting and in activated human T lymphocytes. In addition, we used experimental autoimmune encephalomyelitis (EAE) as a model of T-cell mediated autoimmune disease to assess FK866 efficacy *in vivo*. We show that activated, but not resting, T lymphocytes undergo massive NAD^+^ depletion upon FK866-mediated Nampt inhibition. As a consequence, impaired proliferation, reduced IFN-γ and TNF-α production, and finally autophagic cell demise result. We demonstrate that upregulation of the NAD^+^-degrading enzyme poly-(ADP-ribose)-polymerase (PARP) by activated T cells enhances their susceptibility to NAD^+^ depletion. In addition, we relate defective IFN-γ and TNF-α production in response to FK866 to impaired Sirt6 activity. Finally, we show that FK866 strikingly reduces the neurological damage and the clinical manifestations of EAE. In conclusion, Nampt inhibitors (and possibly Sirt6 inhibitors) could be used to modulate T cell-mediated immune responses and thereby be beneficial in immune-mediated disorders.

## Introduction

FK866 (formerly WK175) is a potent inhibitor of nicotinamide phosphoribosyltrabsferase (Nampt), the key enzyme in the NAD^+^ synthesis pathway from Nam [1], [2], [3]. Initial studies carried out in cancer cell lines indicated that exposure to FK866 results in a slowly progressing form of cell death due to intracellular NAD^+^ depletion [1], [4]. Following preclinical evaluation in animal models, FK866 underwent clinical experimentations in patients with advanced solid tumors showing some activity and acceptable toxicity [5]. Subsequent studies demonstrated that the activity of FK866 is improved when the drug is used in combination with ionizing radiations and with DNA damaging agents as these treatments result in activation of the NAD^+^-degrading enzyme poly-(ADP-ribose) polymerase (PARP) which in turn cooperates to lower NAD^+^ levels in the cell [6], [7], [8].

The preclinical studies and the clinical experimentation revealed that lymphocytes are probably the normal cell type that is most sensitive to FK866 since lymphopenia was consistently observed in response to this drug [5]. In line with these findings, using a mouse strain lacking Nampt expression in the T and B cell lineage, Rongavaux and colleagues have recently shown that Nampt is critically required for the development of both T and B lymphocytes [9]. Finally, T lymphocytes upregulate Nampt in response to mitogenic stimuli [10], [11], [12], [13], suggesting that Nampt activity may be especially required during the process of T lymphocyte activation.

Here, we evaluated FK866 for its capacity to interfere with T lymphocyte function and survival and assessed the mechanisms underlying FK866 efficacy in these cells. We show that Nampt inhibition with FK866 has catastrophic consequences in activated T cells where it virtually obliterates intracellular NAD^+^ stores leading to functional impairment and ultimately to autophagic cell demise. In line with these premises, we show that FK866 strikingly ameliorates the manifestations of experimental autoimmune encephalomyelitis (EAE), a prototypical model of T-cell mediated autoimmune disorder. Our data provide the rational for the evaluation of Nampt inhibitors in immune mediated disorders.

## Results

### FK866 Prevents T Lymphocyte Proliferation and Selectively Affects Viability of Activated T Lymphocytes

**Figure 1:**
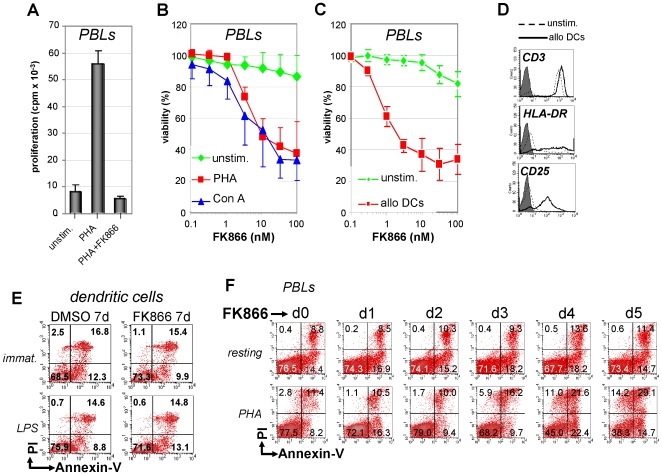
Nampt inhibition with FK866 prevents T lymphocyte proliferation and selectively kills activated T cells. A, PBLs were seeded in 96-well plates in the presence or absence (unstim.) of PHA and 33 nM FK866. Proliferation was assessed 96 h later by standard [^3^H]thymidine incorporation assay. B, PBLs were incubated in 96-well plates in the presence or absence of 5 µg/ml PHA, 1 µg/ml Con A, with or without the indicated concentrations of FK866. Five days later viability was detected by PI staining and flow cytometry. Spontanous cell death was 12.2% and 28.1% for PHA- and Con A-stimulated PBLs, respectively. C, D, PBLs were stimulated for 7 days with or without allogeneic mature DCs before FK866 at the indicated concentrations was added. After 5 days viability was assessed by PI staining and flow cytometric analysis using the lymphocyte gate (C). Spontaneous PBL death was 18.4%. D: phenotype of unstimulated or DC-stimulated PBLs. E, Immature or LPS stimulated DCs were cultured for 7 days with 33 nM FK866 before staining with FITC-conjugated Annexin-V and PI and flow cytometry. F, Resting or PHA-stimulated PBLs were treated with 33 nM FK866 for the indicated times and subsequently stained with FITC-conjugated Annexin-V and PI for flow cytometric analysis. Mean values ± SD of five (B) and three (A, C) different donors are presented. D–F One representative experiment out of three is shown.

The capacity of FK866 to interfere with T lymphocyte responses was initially tested in proliferation assays which showed how this compound virtually abrogates T cell proliferation in response to mitogenic stimulation (Figure 1A and data not shown). Proliferation inhibition was typically accompanied by morphological changes suggestive of cell demise (data not shown). Therefore we assessed whether FK866 would affect T cell viability and whether resting and activated T lymphocytes would behave differently in terms of susceptibility to this drug. We found that FK866 was toxic to PBLs when these were concomitantly activated with mitogens [phytohematoagglutinin-P (PHA), or concanavalin A (Con A)], while unstimulated cells were mostly unaffected (Figure 1B). Similarly, activation with allogeneic DCs also sensitized PBLs to FK866-induced cell demise while unstimulated PBLs were less affected (Figure 1C, D). As detected after a five-day treatment, FK866-induced cell death in activated PBLs was concentration dependent, with EC50s comprised between 1 and 10 nM, and typically reached a plateau starting from 30 nM (Figure 1B, C). In resting PBLs, FK866 EC50 was never reached in the concentration range we used. In experiments where FK866 treatment (33 nM) was extended for up to 12 days, unstimulated PBLs were still >80% viable indicating that resting T cells are mostly resistant to FK866-induced cell death (data not shown). Similarly, FK866 did not affect viability of dendritic cells (DCs) or NK cells even after protracted exposure (Figure 1E and data not shown). Activated T lymphocyte death in response to FK866 was typically a slowly progressing form of cell demise as an increase in the rate of dead T lymphocytes was normally observed starting from 72 h treatment (Figure 1F).

CD3-neg PB mononuclear cells (PBMCs) were less than 20% of the PBL preparations and this percentage further decreased on exposure to T cell mitogens or to allogeneic DCs (<10%) (Figure 1D and data not shown). In order to exclude that T lymphocyte death induced by FK866 was mediated by the contaminating PBMCs, we performed similar experiments using highly pure CD3^+^ cells isolated by magnetic cell sorting (>95%). These tests reproduced the results shown in Figure 1B–C, confirming that FK866 primarily targets activated T lymphocytes (data not shown).

### Activated PBLs Undergo Massive NAD(H) Depletion following Nampt Inhibition

**Figure 2:**
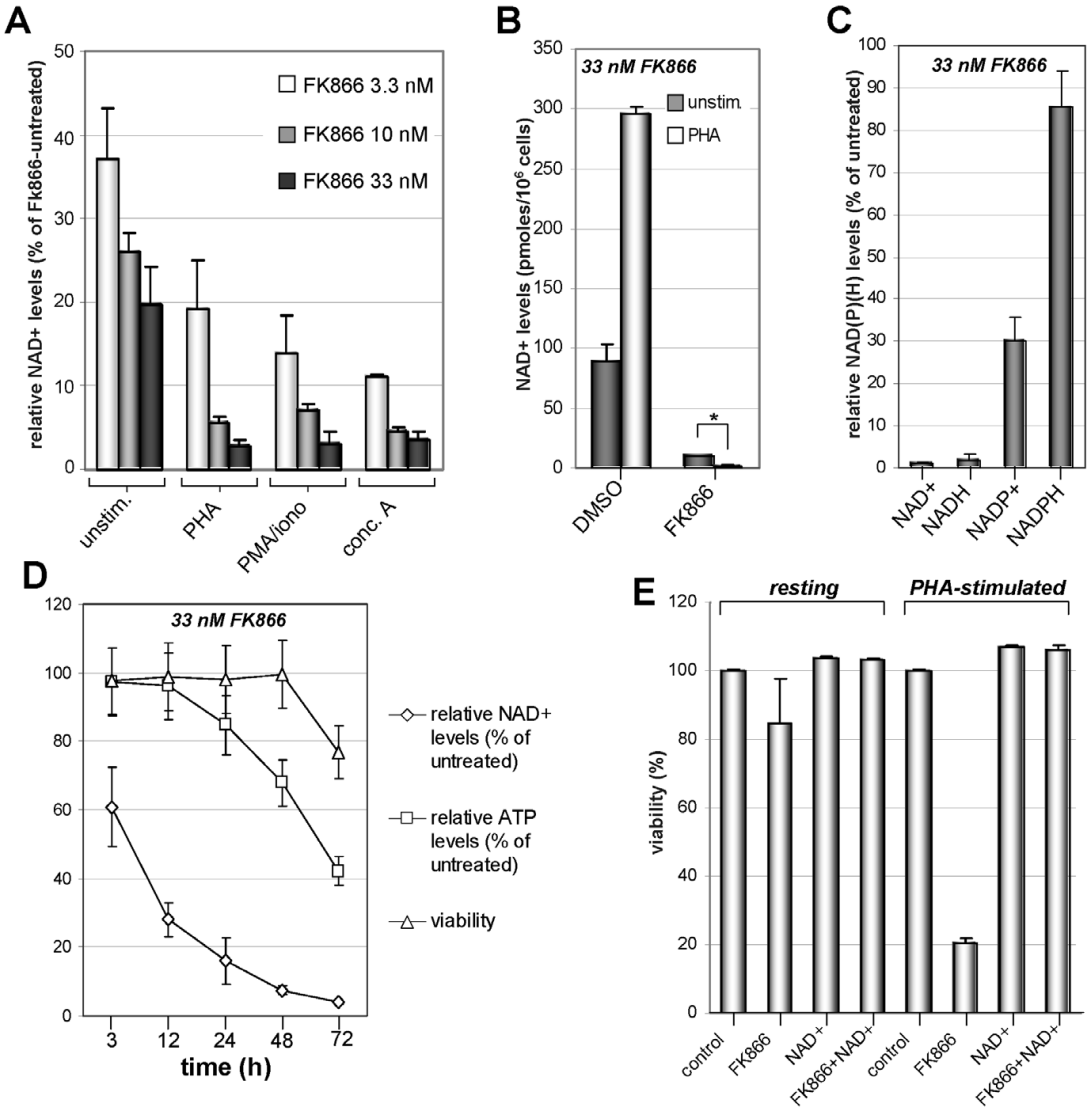
Activated T cells undergo massive NAD^+^ depletion upon Nampt inhibition. A, 3×10^6^ PBLs/well were stimulated (or not, unstim.) with 5 µg/ml PHA, 1 µg/ml con A, or 50 ng/ml PMA and 0.5 µM ionomycin in the presence or absence of the indicated FK866 concentrations. 48 h later, cells were lysed in 0.6 M PCA and NAD^+^ content was measured in neutralized extracts. NAD^+^ levels were normalized to those detected in the absence of FK866. B, Unstimulated or PHA-stimulated PBLs were treated with 33 nM FK866 for 48 h before NAD^+^ content was determined. Absolute NAD^+^ levels are presented. *: p<0.05. C, PBLs were cultured for 48 h with PHA with or without FK866 (33 nM) addition. Subsequently, pyridine dinucleotides levels were measured in acid (NAD^+^ and NAPD^+^) or alkaline (NADH and NADPH) cell extracts. Dinucleotide levels were normalized to those detected without FK866. D, PBLs were incubated with PHA and 33 nM FK866 for the indicated times. Thereafter cells were harvested and NAD^+^ and ATP levels were determined in cell extracts whereas cell viability was assessed by PI-staining and flow cytometry. Results were normalized to the values of FK866-untreated cells. E, Resting or PHA-stimulated PBLs were treated (or not) with 33 nM FK866 in the presence or absence of 1 mM NAD^+^. After five-days viability was assessed determining PI^−^ cells by flow cytometry. Results are means ± SD of five (A) or three (B–E) experiments.

We next sought to determine whether the increased toxicity of Nampt inhibition in activated as compared to resting PBLs would reflect a different response to the drug in terms of intracellular levels of pyridine nucleotides. Intracellular NAD(H) and NADP(H) were measured by cycling enzymatic assays, whereas ATP levels were determined by HPLC. In resting PBLs, the mean NAD^+^, NADH, NADP^+^, and NADPH intracellular amounts were 88±15 pmoles/10^6^ cells, 12.3±0.5 pmole/10^6^ cells, 4.2±0.7 pmoles/10^6^ cells, and 21.7±0.7 pmoles/10^6^ cells, respectively. In mitogen-stimulated PBLs, the mean NAD^+^, NADH, NADP^+^, and NADPH levels were 295.6±5.9 pmoles/10^6^ cells, 60.9±2.1 pmoles/10^6^ cells, 7.1±0.7 pmoles/10^6^ cells, and 94.6±5.6 pmoles/10^6^ cells, respectively. The absolute values of pyridine nucleotides concentrations and their increased content in mitogen-stimulated PBLs are in line with those detected in previous studies [14]. Treatment with FK866 significantly reduced intracellular NAD^+^ levels in both resting and activated T lymphocytes (Figure 2A). However, while unstimulated PBLs retained on average about 20% of their intracellular NAD^+^ content when exposed to 33 nM FK866 (range 11.8%–32%), in mitogen-stimulated T lymphocytes FK866 typically reduced intracellular NAD^+^ to less than 5% of the levels in FK866-untreated cells (range 0.6–7.4%). In activated PBLs treated with FK866, both the absolute NAD^+^ amount and the percentage of NAD^+^ content relative to FK866-untreated controls were significantly lower than those detected in resting PBLs (Figure 2B). NADH levels closely paralleled NAD^+^ levels in response to FK866 (Figure 2C). *Vice versa*, NADP^+^ and particularly NADPH levels were depleted by FK866 to a much lesser extent. Thus, Nampt inhibition appears to primarily affect the NAD(H) pool in T lymphocytes. Figure 2D shows a time course experiment where intracellular NAD^+^ and ATP level, and cell viability were monitored simultaneously in activated PBLs treated with FK866. A significant NAD^+^ decrease was detected as early as 3 h after addition of FK866 (Figure 2D). This was followed by a decrease in intracellular ATP, which became apparent between 12 and 24 h after drug addition. Finally, a decrease in cell viability was detected 72 h after onset of FK866 treatment. These data indicate that T lymphocyte death due to Nampt inhibition is preceded by a period of several hours when cells are viable despite very low NAD^+^ levels and decreasing ATP content. Exogenously added NAD^+^ completely rescued FK866-induced PBL death confirming that the effect of FK866 on PBLs viability is indeed a consequence of NAD^+^-shortage (Figure 2E).

### PARP and Sirtuin Activity Predispose Activated T Lymphocytes to Intracellular NAD^+^ Depletion

**Figure 3:**
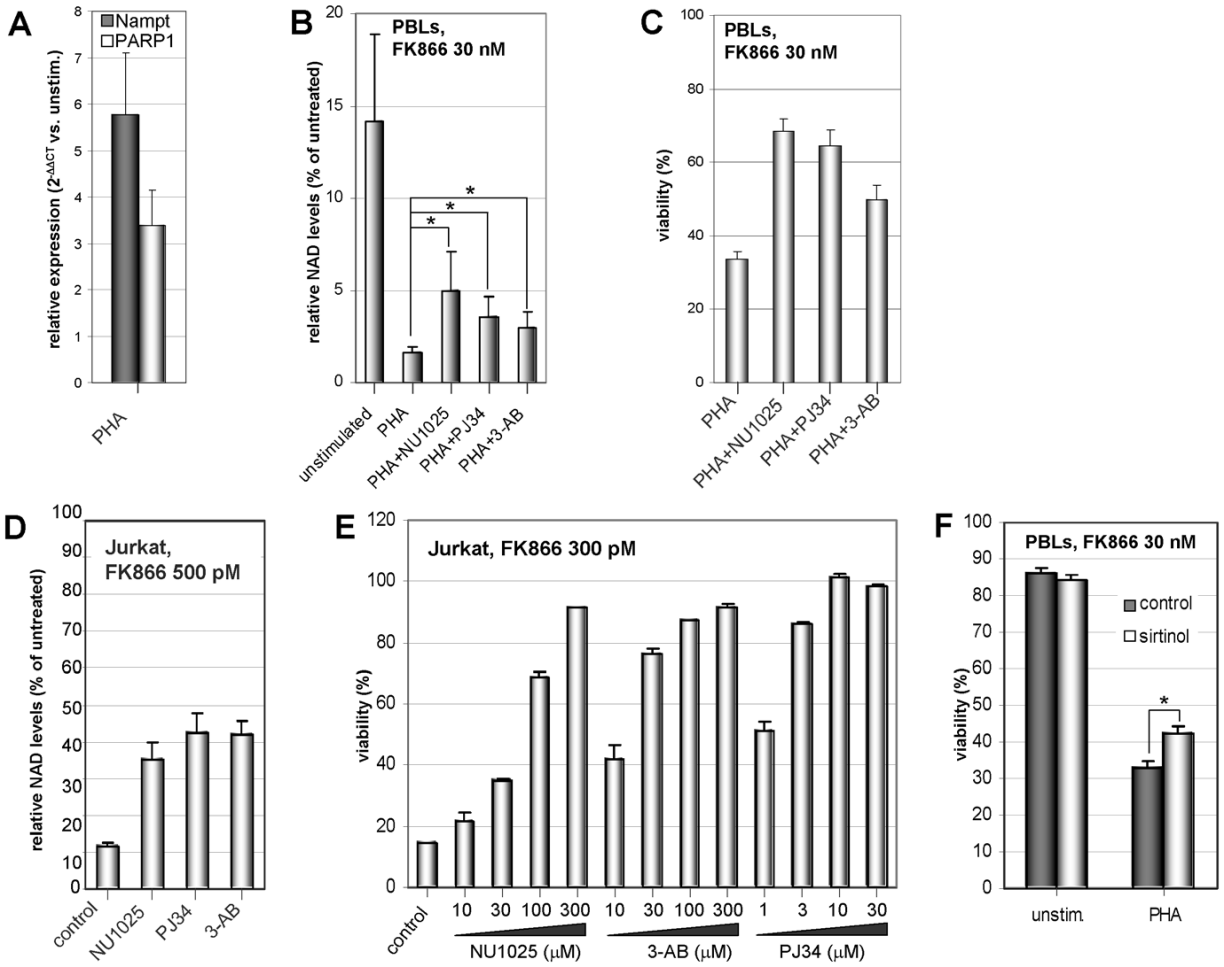
PARP inhibitors and sirtinol attenuate FK866-induced T cell demise. A, PBLs were cultured for 24 h with or without PHA. Thereafter, Nampt and PARP1 levels were detected by Q-PCR. mRNA levels in PHA-stimulated cells were compared to those in unstimulated PBLs. B, Resting or PHA-stimulated PBLs were incubated with or without 33 nM FK866 in the presence or absence of 300 µM NU1025, 10 µM PJ34, or 300 µM 3-AB. 48 h later NAD^+^ levels were assessed (presented as % of values in FK866-untreated PBLs). *, p<0.05. C, PHA-stimulated PBLs were incubated for five days with or without 33 nM FK866 in the presence or absence of 300 µM NU1025, 10 µM PJ34, or 300 µM 3-AB. Thereafter, viability was assessed. D, 5×10^5^ Jurkat cells were treated for two days with 500 pM FK866 in the presence or absence of 300 µM NU1025, 5 µM PJ34, or 300 µM 3-AB. Subsequently, NAD^+^ content was determined and expressed as % of values in FK866-untreated Jurkat. E, 3×10^4^ Jurkat cells/well were incubated in 96-well plates with or without 300 pM FK866 in the presence or absence of the indicated concentrations of NU1025, PJ34, or 3-AB. Viability was determined 96 h later by PI cell staining and flow cytometry. F, PBLs were incubated for five days with PHA, with or without 33 nM FK866, in the presence or absence of 30 µM sirtinol. Viability was subsequently assessed by PI staining and flow cytometry. C, E, F, each treatment was tested in triplicate wells. Results are presented as means ± SD of three experiments.

DNA-damaging agents leading to PARP activation were previously shown to increase FK866 activity [6], [7]. In activated T lymphocytes, a marked increase in PARP activity occurs to ensure efficient DNA repair and possibly to implement signal transduction [10], [15], [16]. Therefore, we assessed whether PARP is involved in conferring susceptibility to FK866 to activated T cells. We first verified by quantitative PCR (Q-PCR) (Figure 3A) and by Western blotting (not shown) that PARP1 and Nampt become upregulated in PBLs in response to mitogenic stimulation. Three structurally unrelated PARP inhibitors, NU1025, 3-AB, and PJ34 indeed provided a partial rescue of intracellular NAD^+^ levels upon treatment of activated PBLs with FK866 (Figure 3B). This effect was paralleled by an increase in cell viability (Figure 3C). NU1025, 3-AB, and PJ34 had no effect on T lymphocyte proliferation in response to mitogens (data not shown). Thus, the protection conferred by these compounds against FK866-induced cell demise is not attributable to inhibition of T lymphocyte activation. In line with a recent report [4], we found that several T cell leukemia cell lines are highly susceptible to FK866 (not shown). In Jurkat cells, NU1025, 3-AB, and PJ34 alleviated FK866-induced NAD^+^ depletion (Fig. 3D) and virtually completely prevented cell death induced by low FK866 concentrations (<500 pM) in a concentration dependent fashion (Figure 3E). However, similarly to what observed in primary PBLs, at higher FK866 concentrations the protection conferred by PARP inhibitors to Jurkat cells and to other T cell leukemia cell lines was only partial (not shown).

The fact that PARP inhibitors only partially block FK866-induced NAD^+^ shortage and cell death in activated T cells suggests that other enzymatic activities/metabolic processes could come into play to enhance susceptibility to FK866. The ADP-ribosyl cyclase CD38 is also upregulated in T cells by activation stimuli [17]. The possible contribution of CD38 to the enhanced sensitivity of activated T lymphocytes to FK866 was first assessed by transfecting Jurkat cells with CD38-specific small interfering RNAs, in the presence or absence of FK866. CD38 silencing did not induce any significant effects on NAD^+^ levels and on viability (data not shown). Moreover, we did not observe a difference in the sensitivity to FK866 between HeLa cells transfected with sense-(CD38^+^) or with anti-sense-(CD38^−^) human CD38 (data not shown) [18].

Finally, sirtuins also use NAD^+^ as a substrate [19], and some of these enzymes may undergo upregulation in activated T lymphocytes (see below). We found that the sirtuin inhibitor sirtinol induced a slight, although significant increase in the levels of intracellular NAD^+^ in activated T cells exposed to FK866 (data not shown). Consistently, sirtinol mitigated FK866-induced cell death in activated PBLs (Figure 3F). Thus, sirtuin activity appears to also be among the factors involved in conferring susceptibility to FK866 to activated T cells.

### NAD^+^ Depletion Induces T Lymphocyte Death via Mitochondria Dysfunction and Autophagy

**Figure 4:**
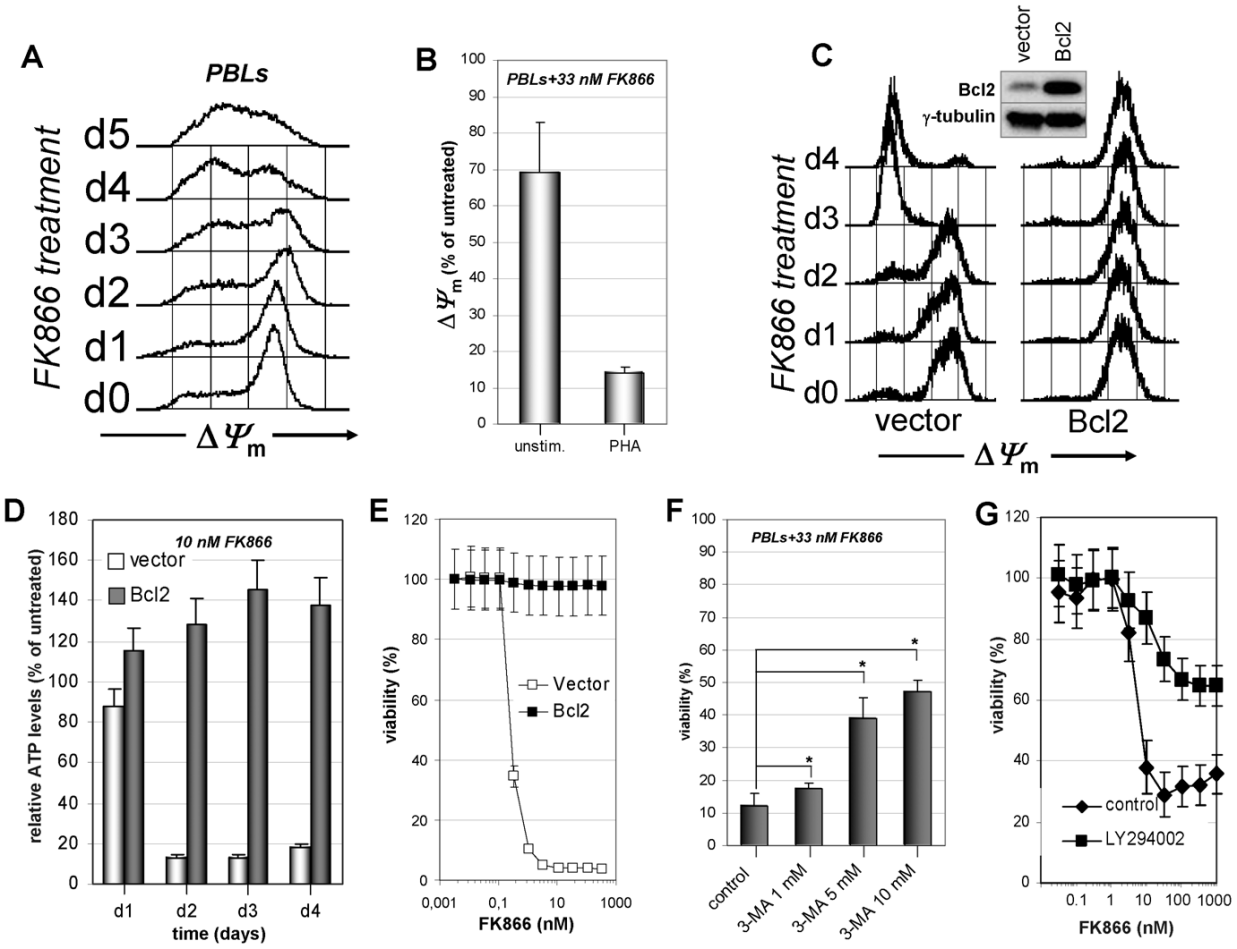
Nampt inhibition with FK866 induces mitochondria depolarization and ATP depletion in activated T lymphocytes. A, PHA- stimulated PBLs were incubated with 33 nM FK866 and *ΔΨ*_m_ was determined at the indicated days of exposure. B, Resting of PHA-stimulated PBLs were cultured with 33 nM FK866 for five days. Thereafter PBLs with conserved *ΔΨ*_m_-high were quantified by flow-cytometry. C, Bcl2-overexpressing Jurkat and the respective vector control cells were incubated with 10 nM FK866 for the indicated number of days. Thereafter, *ΔΨ*_m_ was determined. Inset, Western blot for Bcl2 and γ-tubulin expression. D, 5×10^5^ Bcl2-overexpressing Jurkat and the vector control cells were incubated with or without 10 nM FK866 for the indicated times before ATP was detected. ATP levels are presented as % of ATP in FK866-untreated cells. E, 3×10^4^ Bcl2-overexpressing and control Jurkat cells/well were incubated in 96-well plates with or without the indicated FK866 concentrations. Viability was determined by PI staining and flow cytometry 96 h later. F, PHA-stimulated PBLs were incubated in the presence or absence of 33 nM FK866 with or without the indicated concentrations of 3-MA. Viability was detected after five days. *, p<0.05. G, PBLs incubated in 96-well plates in the presence of 5 µg/ml PHA were treated for five days with the indicated FK866 concentrations in the presence or absence of 20 µM LY294002. Viability was subsequently determined by PI staining and flow cytometry. B, Results are presented as means ± SD of three experiments (B, D–G). Panels A and C are representative of three separate experiments.

Mitochondrial oxidative phosphorylation is the cellular major ATP source under aerobic conditions. NADH is required for mitochondrial transmembrane potential (*ΔΨ*_m_) maintenance which in turn is a prerequisite for ATP generation [20]. We found that in primary activated PBLs as well as in T cell leukemia cell lines *ΔΨ*_m_ is dissipated following FK866-induced NAD^+^ depletion (Figure 4A and data not shown). *ΔΨ*_m_ loss due to Nampt inhibition was more pronounced in activated than in resting PBLs (Figure 4B). To demonstrate that *ΔΨ*_m_ loss is causally involved in FK866-mediated cell death we made use of Jurkat cells engineered to overexpress Bcl2 as this antiapoptotic protein maintains *ΔΨ*_m_ in the presence of *ΔΨ*_m_ loss-inducing stimuli by enhancing H^+^ efflux (Figure 4C) [21], [22]. Indeed, Bcl2-overexpression protected Jurkat cells from FK866-mediated mitochondrial depolarization (Figure 4C). This effect was not due to interference by Bcl2 on intracellular NAD^+^ depletion as FK866 efficiently depleted intracellular NAD^+^ also in Bcl2-overexpressing Jurkat (data not shown). Consistent with the fact that *ΔΨ*_m_ is maintained by Bcl2 overexpression irrespective of NAD^+^ shortage, Bcl2 Jurkat cells did not show ATP depletion upon treatment with FK866 (Figure 4D). Thus, ATP reduction in response to NAD^+^ depletion is due to *ΔΨ*_m_ loss. Finally, Bcl2-overexpressing Jurkat cells were virtually completely resistant to FK866-induced cell death (Figure 4E).

ATP depletion triggers autophagy [23]. To determine the role of autophagy in cell death caused by FK866 we made use of 3-MA and LY294002 two class III phosphoinisitide-3 phosphate kinase (PI3K) inhibitors known to block autophagy [24]. Both inhibitors reduced FK866-induced cell demise in PBLs (Figure 4F, G) as well as in Jurkat cells and in the T cell leukemia cell line H9 (data not shown). No protection from FK866 was conferred by the mitogen-activated protein kinase inhibitor PD098059 or by the NF-κB inhibitor BAY 11–7082 (data not shown). PI3K inhibition in activated PBLs had no effect on PARP upregulation in response to PHA (not shown), ruling out that the PI3K inhibitors may interfere with FK866-induced cell death by affecting PARP. Therefore, in line with previous studies [4], [25], FK866 induces autophagic cell death in activated T lymphocytes.

Since mitochondria dysfunction is also a trigger of the intrinsic apoptotic pathway [26], we assessed whether this mechanism would also contribute to FK866 cytotoxicity. Indeed, we detected released cytochrome *c* in the cytosolic fraction of FK866 treated T lymphocytes (not shown). However, the pan-caspase inhibitor zVAD-fmk, the caspase-9 inhibitor Z-LE(OMe)HD(OMe)-FMK, as well as the caspase-8 inhibitor Z-IE(OMe)TD(OMe)-FMK failed to protect from FK866-induced cell death (data not shown). Thus, although associated with cytochrome *c* cytosolic relocalization, cell demise via NAD^+^ depletion appears to occur in a caspase-independent fashion.

### Nam and Na Prevent NAD^+^ Shortage and Lymphocyte Death Induced by Nampt Inhibition

**Figure 5:**
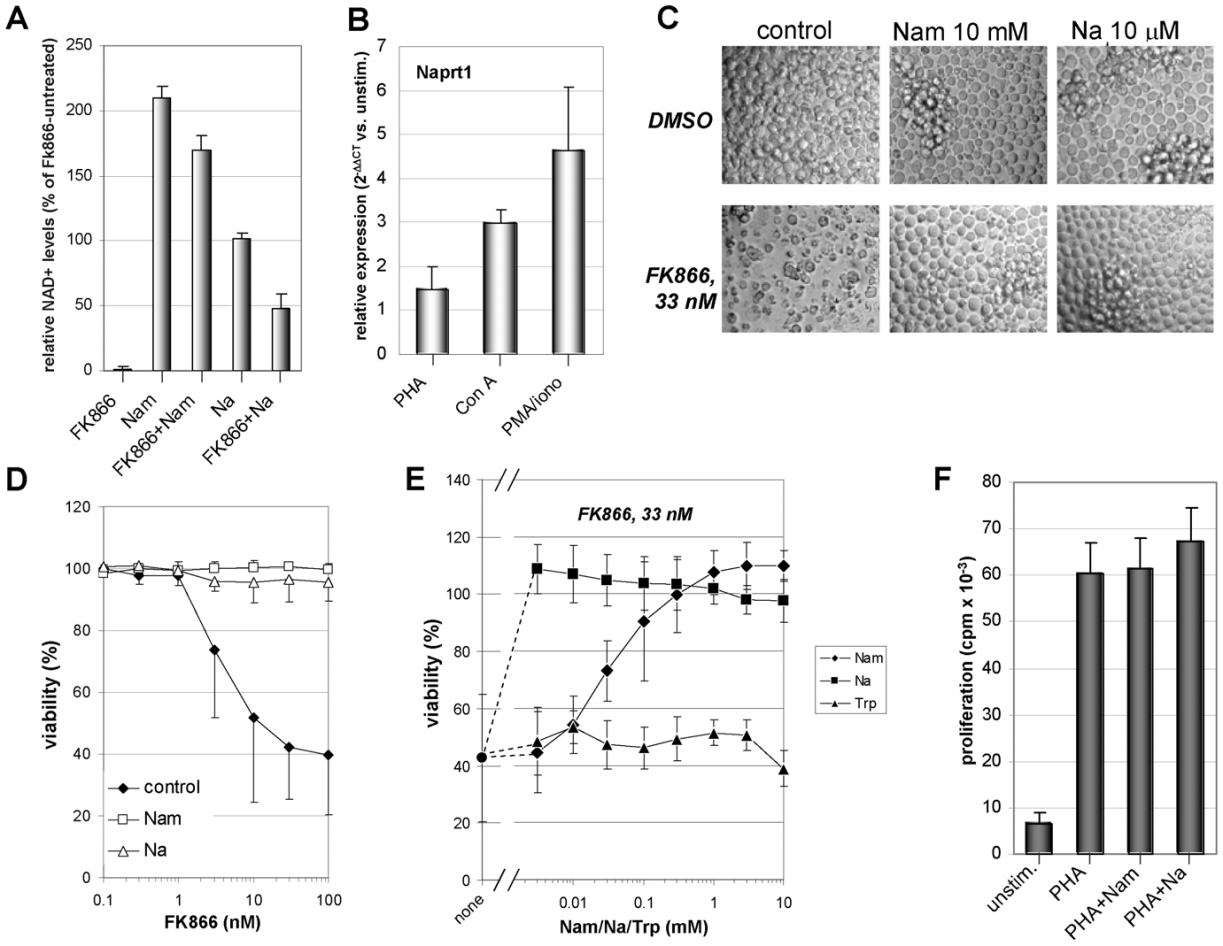
Nam and Na prevent NAD^+^ shortage and cell death induced by FK866 in human T lymphocytes. A, PHA-stimulated PBLs were treated (or not) with 33 nM FK866 in the presence or absence of 10 mM Nam or of 10 µM Na. After 48 h, NAD^+^ content was determined (expressed as percentage of NAD^+^ content in FK866-untreated cells). B, PBLs were cultured for 24 h with or without PHA, 1 µg/ml Con A, or 50 ng/ml PMA and 0.5 µM ionomycin. Thereafter, Naprt1 mRNA levels were detected by Q-PCR. mRNA levels in mitogen-stimulated PBLs were compared to those in unstimulated PBLs. C, D, PHA-stimulated PBLs were incubated for five days with or without 10 mM Nam or 10 µM Na in the presence or absence of the indicated FK866 concentrations. Thereafter, cells were imaged by light microscopy (C), and cell viability was determined (D). E, PHA-stimulated PBLs were incubated for five days with or without 33 nM Fk866 in the presence of the indicated concentrations of Nam, Na, or tryptophan (Trp). Viability was subsequently determined. F, PBLs were stimulated with or without PHA in the presence or absence of 1 mM Nam or Na. Thymidine incorporation was measured after 48 h by a 16-h pulse with 0.5 µCi/well [^3^H]thymidine. D-F each treatment was tested in triplicate wells. Results are means ± SD of three (A, B, F) or four (D, E) experiments. In panel C, one representative experiment out of three is shown.

We performed experiments to evaluate whether FK866-induced NAD^+^ depletion and cell death in PBLs would be prevented by Nam and by Na. Nam is known to act as an antidote for FK866 and recent studies showed that this effect may be due to direct competition for the binding site on the enzyme [1], [2]. Conversely, Na supplementation rescues NAD^+^ synthesis in cells that express Na phosphoribosyltransferase (Naprt1) and can thereby synthesize NAD^+^ via the Preiss-Handler pathway [1], [27]. Nam raised intracellular NAD^+^ levels to over 150% of the untreated control in both FK866-treated and untreated cells (Figure 5A). We verified Naprt1 expression in human PBLs by Q-PCR. Moreover, we found that Naprt1 mRNA is upregulated in activated T cells, with the extent of the upregulation varying depending on the stimulus used (Figure 5B). Na *per se* did not affect intracellular NAD^+^ levels. However, it partially prevented NAD^+^ depletion upon treatment with FK866, to about 50% of the pyridine dinucleotide content in untreated T lymphocytes (Figure 5A). Both Nam and Na efficiently countered PBL death in the presence of cytotoxic FK866 concentrations (Figure 5C) and over a wide range of FK866 concentrations (Figure 5D). However, the potency of Nam and Na as FK866 antagonists was different: Na protected PBLs from 33 nM FK866-induced cell death with an EC50 of 10^−6^ M (Figure 5E, and data not shown) compared to an EC50 value for Nam of 3×10^−5^ M. No protection from FK866-induced cell death was conferred to PBLs by tryptophan supplementation indicating that the de novo NAD^+^ synthesis pathway is not a relevant source of NAD^+^ in human T cells [27]. Finally, Nam and Na did not exert their protective effects by interfering with the process of T lymphocyte activation, since proliferation (Figure 5F) and activation markers (CD25, HLA-DR, not shown) were normally induced in PBLs by PHA regardless of the presence of these metabolites.

These findings are consistent with the occurrence of the Preiss-Handler pathway of NAD^+^ biosynthesis in PBLs, although its contribution to replenish the NAD^+^ pool upon FK866 treatment seems to be limited (see Discussion).

### NAD^+^ Shortage Prevents IFN-γ Production by Activated T Lymphocytes

**Figure 6:**
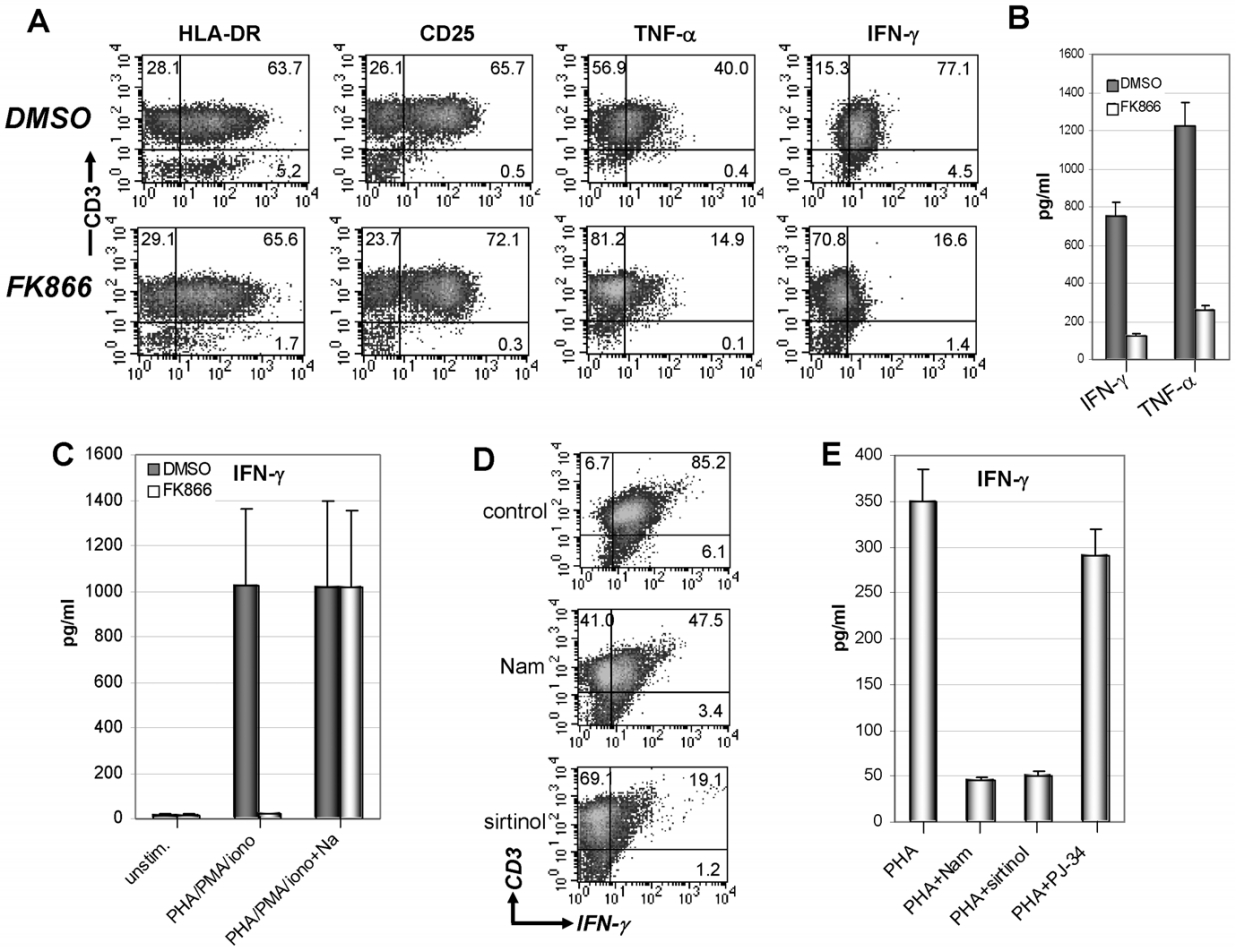
Intracellular NAD^+^ depletion prevents TNF-α and IFN-γ production by activated T lymphocytes. A, B, 5×10^6^ PBLs were stimulated with 5 µg/ml PHA with or without 33 nM FK866 for 36 h and subsequently incubated with 50 ng/ml PMA and 0.5 µM ionomycin for further 5 h. Thereafter, HLA-DR and CD25 expression were detected by flow cytometry. TNF-α and IFN-γ content was determined by intracellular cytokine staining and flow cytometry (A). Released TNF-α and IFN-γ were measured by ELISA (B). DMSO and FK866-treated cells were 84% and 83% viable, respectively. C, 5×10^6^ PBLs were stimulated with PHA and PMA/ionomycin as above, or left unstimulated. Where indicated, 33 nM FK866 with or without 10 µM Na was added. Released IFN-γ was measured by ELISA. Unstimulated PBLs treated with DMSO or FK866 were 96.3% and 96.5% viable, respectively. PBLs stimulated with PHA/PMA/ionomycin and treated with DMSO or FK866 were 89% and 89.5% viable, respectively. PBLs stimulated with PHA/PMA/ionomycin/Na and treated with DMSO or FK866 were 88% and 87% viable, respectively. D, PBLs were stimulated with PHA and PMA/ionomycin as describe for panel A. Where indicated 10 mM Nam or 50 µM sirtinol were added. Thereafter, cells were harvested and intracellular IFN-γ content was determined by flow cytometry. E, PBLs were stimulated with PHA and PMA/ionomycin as above in the presence or absence of 10 mM Nam, 50 µM sirtinol, or 10 µM PJ34. 42 h later, the supernatants were collected and IFN-γ levels were detected by ELISA. Results are means ± SD of four experiments (B, C, E). Panels A and D are representative of four separate experiments.

Nampt inhibition and the consequent NAD^+^ shortage were previously shown to negatively affect secretion of IL-1β, IL-6 and TNF-α in macrophages and dendritic cells [28], [29]. Thus, we performed experiments to determine whether NAD^+^ depletion via FK866 would also affect cytokine secretion in T lymphocytes. These experiments were performed within 48 h from FK866 addition when T cell viability was still unaffected to be able to detect functional changes and avoid the interference of autophagic cell destruction. In this time frame, FK866 had no effect on the upregulation of activation markers such as CD25 and HLA-DR in mitogen-stimulated PBLs (Figure 6A). However, intracellular cytokine staining revealed that FK866 strongly reduced the number of TNF-α-producing CD3^+^ T cells and virtually abrogated IFN-γ synthesis (Figure 6A). These results were confirmed by direct measurement of TNF-α and IFN-γ in the PBL supernatants (Figure 6B). Addition of Na, which replenishes intracellular NAD^+^ stores but does not inhibit sirtuin activity (see below) [30], fully restored IFN-γ synthesis by PBLs (Figure 6C) demonstrating that IFN-γ downregulation was due to NAD^+^ depletion.

Defective TNF-α synthesis as a consequence of NAD^+^ shortage was attributed to reduced function of the NAD^+^-dependent sirtuin Sirt6 [29]. Interestingly, Sirt6-deficient animals develop profound lymphopenia as a result of a cell non-autonomous mechanism, suggesting a possible defect of relevant cytokines or growth factors involved in T cell function [31]. Thus, we evaluated whether IFN-γ secretion inhibition may depend on reduced Sirt6 activity. Indeed, two structurally unrelated sirtuin inhibitors, sirtinol and Nam, prevented IFN-γ expression in T cells as shown by intracellular cytokine staining and IFN-γ detection in the supernatants (Figure 6D, E) confirming a putative role for a sirtuin member in IFN-γ synthesis. Conversely, the PARP inhibitor PJ34 did not affect IFN-γ production (Figure 6E).

**Figure 7:**
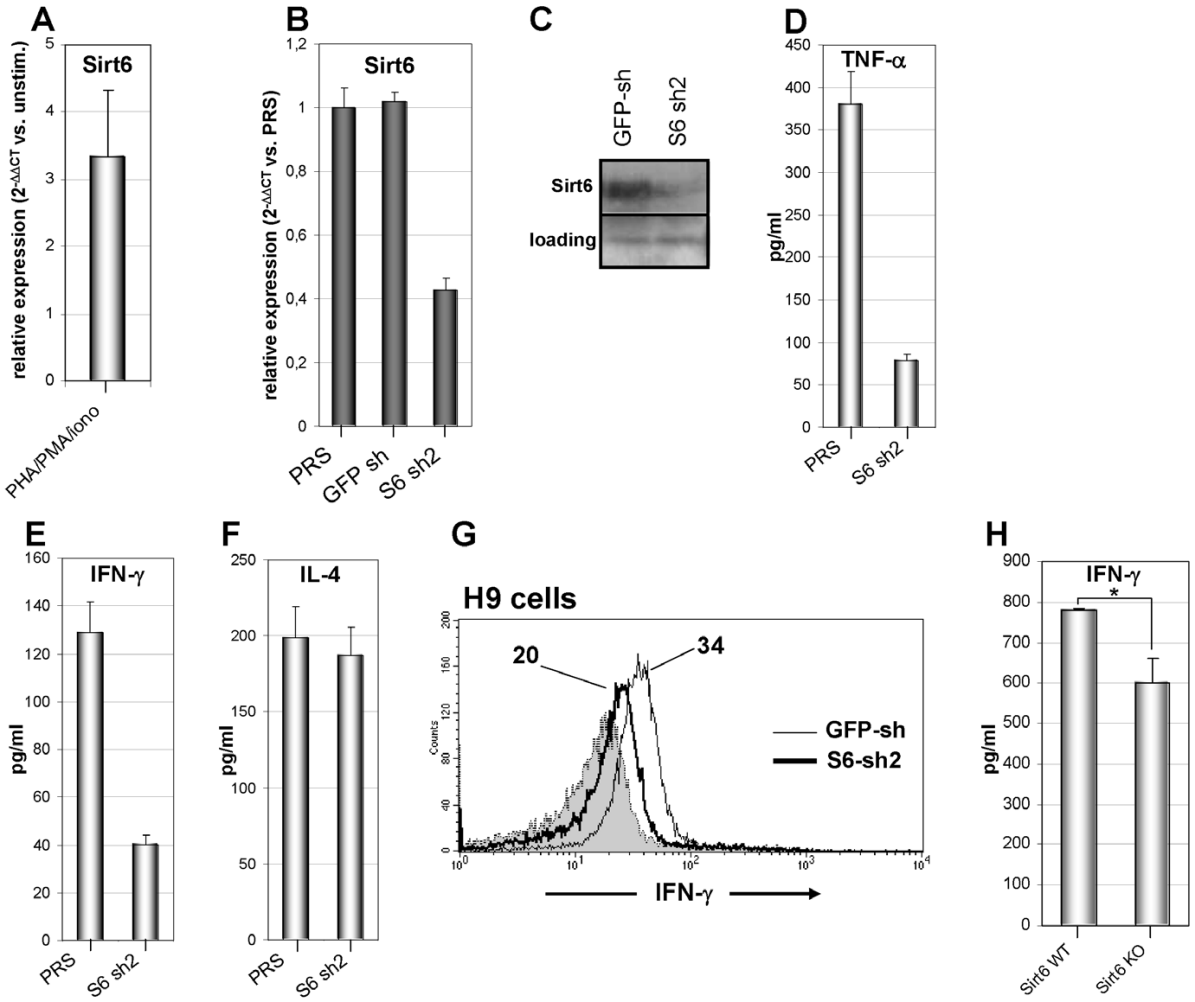
Evidence for an involvement of Sirt6 in IFN-γ synthesis. A, PBLs were cultured for 24 h with or without PHA. Thereafter, Sirt6 levels were detected by Q-PCR. mRNA levels in PHA-stimulated cells were compared to those in unstimulated PBLs. B, C, Jurkat cells were transduced with PRS, (PRS) GFP-sh, or (PRS) S6 sh2, subsequently, Sirt6 mRNA levels or Sirt6 protein levels were determined by Q-PCR (B) and immunoblotting (C). D–F, Jurkat cells transduced with PRS or S6 sh2 were stimulated for 12 h with 5 µg/ml PHA, 50 ng/ml PMA, and 0.5 µM ionomycin. Thereafter, supernatants were harvested and TNF-α (D), IFN-γ (E), and IL-4 (F) levels were determined by ELISA. G, H9 cells transduced with GFP-sh or S6 sh2 were stimulated for 12 h with 5 µg/ml PHA, 50 ng/ml PMA, and 0.5 µM ionomycin. Thereafter, intracellular IFN-γ was detected by intracellular staining. Mean fluorescence intensity for IFN-γ expression is indicated for each histogram. H, 3×10^6^ splenocytes from wild type or *Sirt6 KO* mice/well were seeded in 24 well plates and stimulated for 24 h with 1 µg/ml Con A. Thereafter, supernatants were harvested and IFN-γ levels were determined by ELISA. *: p<0.05. Results are means ± SD of three experiments (A, B, D–F). Panel C and G are representative of three separate experiments.

When evaluating Sirt6 levels in T lymphocytes, we found that Sirt6 transcription is induced by mitogenic stimulation (Figure 7A). To assess the role of Sirt6 in IFN-γ and TNF-α production by T cells we expressed a validated Sirt6 shRNA (S6 sh2) in Jurkat and H9 cells by retroviral transgenesis [32]. Indeed, S6 sh2 effectively downregulated Sirt6 mRNA and protein as shown in Jurkat cells (Figure 7B, C). No detrimental effect of Sirt6 silencing on cell viability was observed (data not shown). Jurkat cells with reduced Sirt6 levels showed reduced IFN-γ and TNF-α production on stimulation with mitogens (Figure 7D, E). Conversely, IL-4 secretion was not affected by Sirt6 deficiency (Figure 7F). Sirt6 silencing led to reduced intracellular IFN-γ levels in H9 cells stimulated with a mitogen cocktail (Figure 7G). Finally, splenocytes from Sirt6 knockout mice were found to secrete less IFN-γ than splenocytes from wild type animals (Figure 7H). Thus, altogether these data are consistent with an involvement of Sirt6 in IFN-γ synthesis.

### FK866 Reduces Demyelination and Disease Severity in EAE

**Figure 8:**
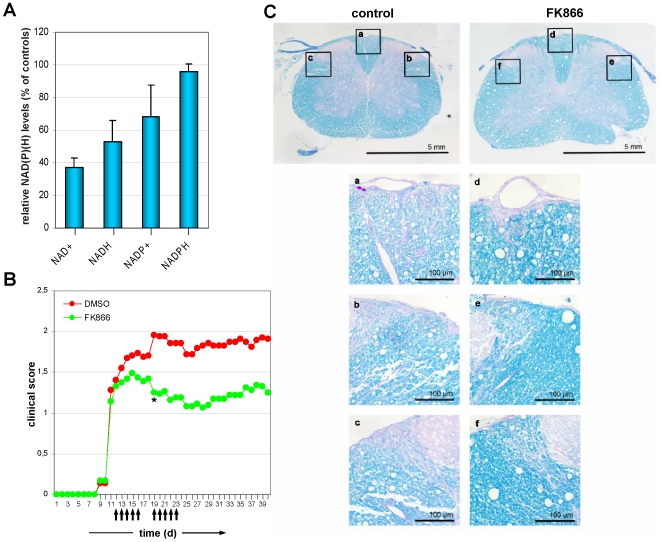
FK866 ameliorates EAE. 10 mg/kg body weight FK866 were administered to mice from day 12 after rMOG immunization for 10 days. A, NAD(H) and NADP(H) levels were measured in mononuclear cells isolated from spleen and lymph nodes of treated or untreated animals at day 16. Dinucleotide levels in FK866-treated mice were expressed as % of those detected in control animals. B, FK866 halts EAE severity compared with controls (p<0.05 from day 19 onward). Arrows indicate the days of FK866 administration. C, Luxol fast Blue staining of the spinal cord shows areas of demyelination in control mice compared with FK866-treated animals.

Given the capacity of FK866 to interfere with survival of activated T cell and with the release of immunogenic cytokines, we sought to determine whether FK866 treatment might be advantageous in a prototypical autoimmune disease such as EAE, particularly after disease onset. FK866 was administered at 10 mg/Kg body weight twice daily starting 12 days post MOG-immunization of the mice, for a total of 10 days. Mononuclear cells from FK866-treated animals exhibited a significant decrease in the levels of intracellular pyridine dinucleotides (Figure 8A). As observed with human PBLs, NAD^+^ and NADH were more severely reduced as compared to NADP^+^ and NADPH. FK866 strikingly reduced the clinical disease score as described in Materials and Methods when compared with vehicle-alone treated controls, reaching statistical significance from day 19 onward (Figure 8B, and Table 1). The benefit of FK866 was fully retained until day 80 from immunization (not shown). The clinical effect of FK866 was associated with a marked reduction of demyelination in the spinal cord of treated mice (Figure 8C, and Table 1). FK866 showed no evident toxicity on renal and liver function, nor did FK866-treated mice show weight loss or increased frequency of concomitant infectious or neoplastic disease over a period of 80 days. Hence, FK866 is effective in treating EAE after disease onset in the absence of obvious toxicity.

**Table 1:** Clinical-pathologic features of EAE-affected mice treated with FK866.

	*Disease incidence no./no. total (%)*	*Disease onset, day after immunization*	*Mean maximum neurologic score*	*Cumulative disease score*	*Demyelination score*
***Control***	16/16 (100%)	10.5±0.9	2.4±0.9	53.9±23.6	10.6±4.6
***FK866***	16/16 (100%)	10.3±0.9	1.9±0.8	37.7±22.4*	8.1±3.6*

*p<0.05 (Mann-Whitney test).

## Discussion

In this study, we show that activated T lymphocytes are crucially dependent on Nampt activity for their function and survival as they face massive NAD^+^ depletion and cell demise when this enzyme is obstructed with FK866. Thus, increased susceptibility to NAD^+^ lowering drugs emerges as a property of activated T lymphocytes that is susceptible of being targeted pharmacologically. The consequences of Nampt inhibition in unstimulated T lymphocytes appear to be less deleterious as these retain NAD(H) levels that are sufficient to ensure survival in the majority of the cells.

The high susceptibility of activated T cells to FK866 is due, at least in part, to PARP upregulation which *per se* leads to intracellular NAD^+^ reduction, an occurrence normally prevented by Nampt [8], [10], [12], [16]. The involvement of other enzymatic activities/metabolic processes in enhancing T cell susceptibility to FK866 is suggested by the fact that PARP inhibitors only partially protect activated T lymphocytes from FK866. Experiments aimed to assess the role of the ADP-ribosyl cyclase CD38 in conferring susceptibility to FK866 failed to detect major such effects of this enzyme. Conversely, a partial protection from FK866 was conferred to activated T cells by the sirtuin inhibitor sirtinol. Which one(s) of the sirtuin is (are) most active in T cells, and which role they play has to be defined yet. Nonetheless, our findings suggest that sirtuins may be responsible for consuming significant amounts of NAD^+^ in T lymphocytes. In addition, sirtuin levels/activity may increase in response to activation stimuli, as is the case of Sirt6, which we found to be upregulated in response to mitogens. Finally, since a 5–13-fold increase in NAD^+^ kinase activity was detected in mitogen-stimulated T lymphocytes [10], another mechanism contributing to the exhaustion of the NAD(H) stores in activated T lymphocytes could be the shift of NAD^+^ into the NADP(H) pool by the sequential action of NAD^+^ kinase and glucose-6-phosphate dehydrogenase [33]. In line with this hypothesis is the finding that NADPH levels are virtually unaffected by FK866 in activated T cells while NAD^+^ and NADH virtually disappear. Why other immune cell types are not affected by FK866 as much as activated T lymphocytes are remains a speculative matter. Its is conceivable that the enzymatic activities that actively consume NAD^+^ and that we found upregulated in activated T cells may not be as much represented in other immune cells. Alternatively, other cells of the immune system may obtain their NAD^+^ supplies (also) through metabolic pathways that are not affected by FK866. For instance, it is well documented that activated DCs upregulate indoleamine 2, 3-dioxygenase (IDO), a key enzyme in the NAD^+^ synthesis pathway from tryptophan [27], [34].

T lymphocyte death due to NAD^+^ exhaustion involves *ΔΨ*_m_ dissipation, ATP shortage, and autophagy. Previous studies suggested that FK866 induces apoptosis via cytochrome *c* release and caspase-9 activation [3]. In FK866-treated T lymphocytes, we did detect released cytochrome *c* in the cytosol (not shown). However, different caspase inhibitors failed to protect activated T lymphocytes from FK866 cytotoxicity. Thus, T cell death via FK866, although accompanied by cytochrome *c* release, is caspase-independent and is therefore not a classical apoptotic process.

In addition to the Nam salvage pathway, NAD^+^ synthesis from Na is apparently also an option for T lymphocytes. Previous studies found that Naprt1 activity is present in human PBLs [14]. We confirmed Naprt1 expression in human PBLs by Q-PCR and showed that this enzyme undergoes upregulation upon cell stimulation with mitogens. Consistently, we found that Na supplementation rescues NAD^+^ levels when Nampt is inhibited allowing for T lymphocyte survival. The fact that FK866 induces lymphopenia in patients indicates that, under normal conditions, sufficient Na levels are not available to rescue T lymphocytes *in vivo* [5]. On the other hand, the consequences of Nampt inhibition could theoretically be rescued by increasing dietary Na intake or by pharmacological Na administration [27]. The expression in T lymphocytes of other enzymatic activities involved in NAD^+^ synthesis from NR and NaR remains unexplored and could provide insights into possible ways to modulate FK866 immunosuppressive activity and/or into mechanisms of resistance to this drug by cancer cells.

**Figure 9:**
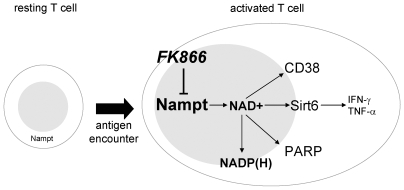
A putative model of Nampt's role in activated T lymphocytes. Nampt activity is responsible for providing sufficient NAD^+^ supplies during T cell activation. NAD^+^, in turn, is required for ATP synthesis, metabolic reactions, and to replenish NADPH levels. In addition, NAD^+^ represents the substrate of NAD^+^-degrading enzymes such as PARP, CD38, and the sirtuins. Among these, Sirt6 appears to have a central role in IFN-γ and TNF-α production. Nampt inhibitors such as FK866 (and possibly Sirt6 inhibitors) could be used to modulate T cell-mediated immune responses and thereby be beneficial in immune disorders.

Not only NAD^+^ shortage has deleterious repercussions on T cell viability. Before dying, T lymphocytes experience a phase of functional impairment where cytokine secretion is prohibited. In addition to reducing TNF-α production as described previously [28], [29], we found that FK866 also abolishes IFN-γ expression. Importantly, in analogy to TNF-α [29], our data are consistent with an involvement of Sirt6 also in IFN-γ synthesis, since RNAi-mediated Sirt6 removal reduces the expression of this cytokine. Moreover, splenocytes from *Sirt6 KO* mice were found to secrete less IFN-γ than cells from wild type mice do. How Sirt6 is involved in TNF-α and IFN-γ production remains to be determined. Nonetheless, taken together, these observations depict the Nampt-Sirt6 axis as an important regulatory pathway for cytokines involved in inflammation and cell-mediated immunity, that could be blocked with FK866 or, possibly, with Sirt6 inhibitors (Figure 9).

Based on these findings, we administered FK866 with the aim of curing EAE, a prototypical T cell-mediated autoimmune disease. Indeed, administration of FK866 after EAE onset successfully ameliorated the severity of disease. The clinical efficacy was demonstrated by a decreased cumulative disease score and by a consistent reduction in demyelination. Our data with FK866 in EAE are in line with recent reports of an anti-inflammatory activity of this drug in carrageenan-induced arthritis [28]. In fact, we cannot exclude that the benefit of FK866 on the clinical and histological manifestations of EAE may derive, at least in part, from effects that are unrelated to its activity on T cells. For instance, FK866 could (also) alleviate EAE by inhibiting TNF-α secretion by macrophages and microglia, thereby reducing inflammation and thus preventing neurological damage [28], [29].

In conclusion, Nampt upregulation during T cell activation appears as an essential adaptation ensuring that sufficient NAD^+^ levels are available for metabolic reactions as well as for NAD^+^-utilizing enzymes involved in DNA repair, signal transduction, and cytokine production. Targeting this metabolic pathway could represent a novel strategy to selectively eliminate activated T cells and block detrimental immune/inflammatory reactions such as those underlying autoimmune diseases, graft-versus-host disease, and transplant rejection.

## Materials and Methods

### Cell Lines and Reagents

The T cell leukemia cell lines Jurkat, H9, PEER, and Phoenix were obtained from ATCC (LGC Standards s.r.l. Milan, Italy). Cells were grown in RPMI 1640-based medium supplemented with 10% FBS and antibiotics. The Bcl-2 overexpressing Jurkat cells and the respective vector control cells were a gift of Dr. Claus Belka (Department of Radiation Oncology, University of Tuebingen, Tuebingen, Germany) [22]. HeLa cells transfected with the sense (CD38^+^) and antisense (CD38^−^) cDNA for human full-length CD38 were obtained and cultured as described [18]. Phytohematoagglutinin-P (PHA), concanavalin A (Con A), ionomycin, PMA, Nam, Na, Trp, 3-methyladenine (3-MA), sirtinol, LY294002, NU1025, 3-AB, PJ34, lipopolysaccharide (LPS), BAY 11–7082, PD098059, and tetramethyl rhodamine ethyl ester (TMRE) were all obtained from Sigma-Aldrich (Sigma Aldrich Italia, Milano, Italy). FK866 was generously provided by the NIMH Chemical Synthesis and Drug Supply Program.

### Peripheral Blood Lymphocyte (PBL), NK Cell, and Dendritic Cell (DC) Isolation

Peripheral blood mononuclear cells (PBMCs) were isolated from blood samples obtained from healthy donors by Ficoll Hypaque density gradient centrifugation. 10^7^ mononuclear cells/well were plated in 6-well plates for 2 h in X-VIVO 20 medium (Bio-Whittaker, Lonza, Milan, Italy) to allow monocytes to adhere. Non-adhering mononuclear cells (>80% CD3+ lymphocytes, PBLs) were harvested by washing with phosphate-buffered saline (PBS, Invitrogen Italia, Milan, Italy). For DC generation, adhering monocytes were cultured in RPMI 1640 medium supplemented with 10% FBS and antibiotics in the presence of 100 ng/ml GM-CSF and 20 ng/ml IL-4 (R&D Systems, Wiesbaden, Germany) [35]. Where indicated, 100 ng/ml LPS were added to induce DC maturation. DC phenotype was confirmed by monitoring CD1a, CD83, HLA-DR, CD80, CD86 and CD14 levels by flow cytometry. NK cells were isolated as described elsewhere [36].

### Immunostaining

Cells were stained using FITC- or PE-conjugated mouse mAbs against CD3, CD25, HLA-DR (BD) and mouse IgG isotype control and analysed using a FACSCalibur (Becton Dickinson Italia, Milan, Italy). For intracellular TNF-α and IFN-γ staining, cells were initially stained with a mouse monoclonal anti-CD3 (FITC- or PE-conjugated). Thereafter, cells were washed and incubated with 0.25% saponin and a FITC-conjugated anti-human IFN-γ or a PE-conjugated anti-human TNF-α (both from Becton Dickinson). Finally, cells were washed and analysed by flow cytometry.

### Viability Assays

2.5×10^5^ PBLs/well were plated in 96 well plates in the presence or absence of the indicated stimuli. Viability was determined 120 h later by propidium iodide (PI) staining and flow cytometry (FACS Calibur, Becton Dickinson, BD Italia, Milan, Italy) within the lymphocyte gate. Viability was calculated with the following formula: 100-[(experimental death-spontaneous death)/(100- spontaneous death)x100]. When DCs were used as a stimulator, 2.5×10^5^ PBLs/well were plated in 96 well plates with 2.5×10^4^ irradiated (3000 rad) allogeneic DCs. FK866 was added at the indicated concentrations 7 days later. For Annexin-V/PI staining, 3×10^6^ PBLs/well were plated in 1 ml medium in 24-well plates in the presence or absence of 5 µg/ml PHA and treated with 33 nM FK866 for the indicated amounts of time. Afterwards, cells were washed, stained with Annexin-V-FITC (Becton Dickinson) and PI and analyzed by flow cytometry.

### Light Microscopy

Cells were imaged at room temperature using the 40× magnification of a Zeiss AXIOVERT200 microscope, camera Qlympus C-4040ZOOM. The image files were acquired with the software Olympus CAMEDIA Master 2.5, and subsequently processed using Microsoft Photo Editor.

### Proliferation Assay

PBLs were seeded at 2×10^5^ per well in 96-well plates. Proliferation was induced by adding PHA (5 µg/ml) and measured after 72 h by a 16-h pulse with [^3^H]thymidine (0.5 µCi/well; Amersham Life Science; Buckingham, U.K.).

### Mitochondrial Transmembrane Potential (*ΔΨ*_m_) Determination

*ΔΨ*_m_ was determined as previously described [37].

### ELISA for Detection of IFN-γ and TNF-α

Supernatants harvested on day 2 from PBLs cultured in the presence of the indicated stimuli were analysed for the content of either IFN-γ or TNF-α by ELISA kits from Peprotech Inc. (Princeton Business Park, Rocky Hill, NJ) according to manufacturer's instructions. IFN-γ secretion by splenocytes isolated from wild type and *Sirt6 KO* mice (16-days old) [31] was detected using a sandwich ELISA kit purchased from R&D Systems Inc. (Minneapolis, MN USA). Data are referred to a standard curve obtained with recombinant IFN-γ or TNF-α, respectively.

### Determination of the Intracellular NAD(H), NADP(H) and ATP Levels

PBLs, resting or activated, were cultured in the presence or absence of FK866: cells were harvested and lysed with 0.6 M perchloric acid (PCA) or 0.1 M NaOH, to determine the content of NAD^+^ or NADH, and NADP^+^ or NADPH, respectively. The alkaline extracts were incubated at 70°C for 10 min. Both acid and alkaline extracts were neutralized and the intracellular content of the various coenzymes was assessed with a sensitive enzyme cyclic assay, which exploits the use of alcohol dehydrogenase or of G6PD, to determine NAD(H) [38], or NADP(H) [39], respectively. To evaluate the content of ATP, cells were lysed in PCA and the neutralized extracts were analyzed by HPLC [40]. NAD(H) and NADP(H) levels in murine mononuclear cells (MNCs) isolated from mice treated (or not) with FK866, were determined as in human PBLs. NAD(H), NADP(H), and ATP values were normalized to protein concentrations (micro-BCA kit, Pierce).

### siRNA Transfection

Jurkat cells were transfected using the Nucleofector System (Amaxa GmbH, Cologne, Germany), without (control), or with StealthTM duplex short interference RNA (siRNA) targeting CD38. Cells were transfected in parallel with StealthTM Negative Control. After transfection, cells were incubated in the presence or absence of FK866 and intracellular NAD+ content was evaluated as described above. To confirm CD38 silencing, CD38 mRNA was detected by quantitative real-time PCR.

### Retroviral Transgenesis

Empty pRETROSuper (PRS) and PRS GFP-sh were from Dr. Thijn Brummelkamp (Whitehead Institute for Biomedical Research, Cambridge, Ma, USA); PRS S6 sh2 was from Dr. Katrin F. Chua (Department of Medicine, Stanford University School of Medicine, Stanford, CA 94305, USA) [32]. Phoenix cells were plated in 4 ml medium in 6 cm-dishes and allowed to adhere for 24 h. Thereafter, cells were transfected with 4 µg plasmid DNA using Transit 293 (Mirus Bio, Madison, WI, USA) according to the manufacturer's instructions. The viral supernatant was harvested 36 and 48 h later and used to infect Jurkat and H9 cells in 24-well plates in the presence of 5 µg/ml protamine sulfate. Successfully infected cells were selected using 1 µg/ml puromycin.

### Q-PCR

Total RNA was extracted from 5×10^5^ cells using RNeasy kit reagents (Qiagen, Qiagen Italia, Milan, Italy). Total RNA (1 µg) was reverse transcribed using random hexamers in a final volume of 50 µl. 5 µl of the resulting cDNA was used for Q-PCR using a TaqMan 7900 HT Fast Real TimeAB [41]. Pre-designed primers and probes for Nampt, PARP1, CD38, Naprt1, and 18S were obtained from Applied Biosystems. Gene expression was normalized to housekeeping gene expression (18S). Comparisons in gene expression were done using the 2^−ΔΔCt^ method [42].

### EAE Induction and Treatment Protocol with FK866

This study was performed in compliance with the US Department of Health and Human Services Guide for the Care and Use of Laboratory Animals and approved by the Internal Review Board of the Advanced Biotechnology Center (ABC) in Genoa, Italy. Female C57BL/6J mice, 6 to 8 weeks old, were purchased from Harlan Italy (S. Pietro al Natisone, Italy). EAE was induced by MOG35–55 immunization according to a previously published protocol [43]. Mice received two intraperitoneal injections daily of 10 mg FK866/kg body weight or DMSO dissolved in 0.5 ml PBS from day 12 through 16, and from day 19 through 23. Groups of 16 mice for each treatment (FK866 vs. DMSO) were used. Weight and clinical score were recorded daily. Clinical scores were assigned according to a standard and validated 0 to 5 scale [43]. Unless moribund, mice were followed for at least 40 days following immunization. Disease incidence, onset, and maximum score were recorded for each mouse and expressed as mean +/−SD. The cumulative disease score was calculated by summing the neurologic scores recorded daily for each mouse along the whole period of observation.

### Histology

Euthanized mice were transcardially perfused with 4% paraformaldehyde before spinal cords were collected and embedded in paraffin. 5-µm sections were stained with hematoxylin and eosin for detection of inflammatory infiltrates while Luxol fast Blue was used to observe myelin. All sections were analyzed with an Olimpus Provis AX70 (Olimpus Italia, Segrate, Milan, Italy) optical microscope. The areas of spinal cord demyelination were identified on individual images and traced manually on the composite images. Their surface was determined with Image Pro-PLUS 4 software (Media Cyberbetics, Silver Spring, MD) and expressed as a percentage of the total surface area. All histological evaluations were performed in a blind fashion.
